# Mouse Age Matters: How Age Affects the Murine Plasma Metabolome

**DOI:** 10.3390/metabo10110472

**Published:** 2020-11-19

**Authors:** Patrick Pann, Martin Hrabě de Angelis, Cornelia Prehn, Jerzy Adamski

**Affiliations:** 1 Research Unit Molecular Endocrinology and Metabolism, Helmholtz Zentrum München, German Research Center for Environmental Health (GmbH), 85764 Neuherberg, Germany; patrick.pann@helmholtz-muenchen.de (P.P.); prehn@helmholtz-muenchen.de (C.P.); 2 German Mouse Clinic, Institute of Experimental Genetics, Helmholtz Zentrum München, German Research Center for Environmental Health (GmbH), 85764 Neuherberg, Germany; hrabe@helmholtz-muenchen.de; 3 German Center for Diabetes Research (DZD), 85764 Neuherberg, Germany; 4 Chair of Experimental Genetics, School of Life Science, Weihenstephan, Technische Universität München, 85354 Freising, Germany; 5 Department of Biochemistry, Yong Loo Lin School of Medicine, National University of Singapore, Singapore 117597, Singapore

**Keywords:** age, metabolism, metabolomics, mouse strain, mouse development

## Abstract

A large part of metabolomics research relies on experiments involving mouse models, which are usually 6 to 20 weeks of age. However, in this age range mice undergo dramatic developmental changes. Even small age differences may lead to different metabolomes, which in turn could increase inter-sample variability and impair the reproducibility and comparability of metabolomics results. In order to learn more about the variability of the murine plasma metabolome, we analyzed male and female C57BL/6J, C57BL/6NTac, 129S1/SvImJ, and C3HeB/FeJ mice at 6, 10, 14, and 20 weeks of age, using targeted metabolomics (BIOCRATES Absolute*IDQ*™ p150 Kit). Our analysis revealed high variability of the murine plasma metabolome during adolescence and early adulthood. A general age range with minimal variability, and thus a stable metabolome, could not be identified. Age-related metabolomic changes as well as the metabolite profiles at specific ages differed markedly between mouse strains. This observation illustrates the fact that the developmental timing in mice is strain specific. We therefore stress the importance of deliberate strain choice, as well as consistency and precise documentation of animal age, in metabolomics studies.

## 1. Introduction

Metabolomics is the study of small-molecule compounds, called metabolites, which are the substrates, intermediates, and products of metabolic reactions. The entire set of metabolites in a biological system is termed the metabolome. As part of the “omics” sciences, metabolomics adds a layer of information to our understanding of the structure and dynamics of biological systems and helps to bridge the genotype-phenotype gap. Metabolomics analysis allows for a snapshot view of the status of an organism at a specific point in time, reflecting the status of the genome, transcriptome, and proteome, as well as their interactions. Metabolomics has developed into a powerful tool in systems biology, and has found its way into disease research, diagnostics, and drug development [1,2]. Progress in these fields relies largely on animal experiments, with mice being the most frequently used species [3]. However, factors such as circadian rhythm, stress, and nutrient intake, as well as the type of anesthesia or euthanasia used are known to influence the outcomes of metabolomics experiments in rodents [4,5,6,7]. Disregarding these factors increases sample variability, which may lead to false negative results, and impairs the reproducibility and comparability of metabolomics studies. Awareness and consideration of confounding factors during experimental design, as well as precise documentation, are key to avoiding these pitfalls.

Animal age is another variable that can influence the murine metabolome, as exemplified by the changes apparent in senescent mice [8,9,10]. However, while mice also undergo extensive physiological changes during postnatal development, little is known about variability in the metabolome during this early stage of life [11,12,13]. According to a survey conducted among scientists from academia and industry, mice are most often used at a very young age regardless of the field of research [14]. Choice of age was found to be often based on considerations like cost, time, or comparability with historical data. Among the mouse models reported as ‘adult’, the ages of the mice ranged from 6 to 20 weeks. These findings suggest that age choice is not only inconsistent but is often not based on murine development.

To investigate the influence of age on the plasma metabolome, we performed targeted metabolomics analysis in male and female mice at different time points between 6 and 20 weeks of age. To obtain a broad picture, we analyzed four mouse strains frequently used in research, C57BL/6J (B6J), C57BL/6NTac (B6NTac), 129S1/SvImJ (129S1), and C3HeB/FeJ (C3Fe). B6J is the most widely used among all inbred mouse strains, while B6NTac is the preferred C57BL/6 substrain of the Knockout Mouse Project (KOMP) and the International Mouse Phenotyping Consortium (IMPC). Strain 129S1 is commonly used as a control for transgenic mouse models created with 129 ‘steel’-derived ES cell lines. C3Fe is mostly used in immunological research.

## 2. Results

### 2.1. Metabolomic Changes during Adolescence and Early Adulthood

**Figure 1:**
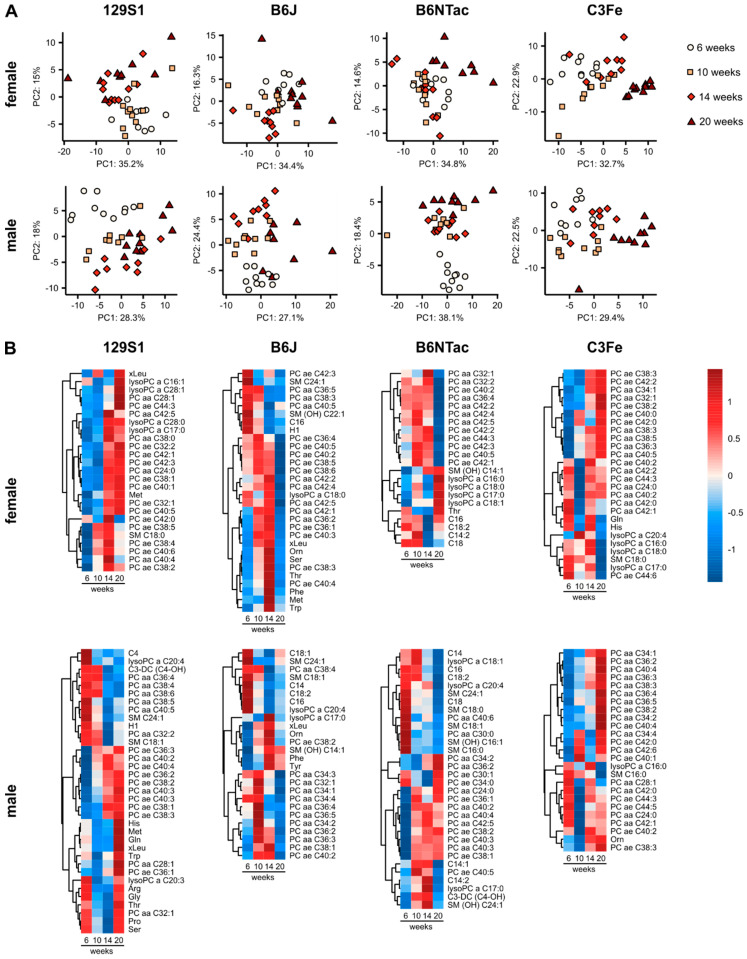
Age-dependent metabolomic changes in murine plasma. (**A**) PCA of metabolomics data at 6, 10, 14, and 20 weeks of age. (**B**) Heatmaps of z scores of top metabolites distinguishing between age. Metabolites were selected based on loading scores of principle components 1 to 3 in PCA and the mean decrease in accuracy in random forest analysis. Z scores were calculated from scaled and centered mean metabolite concentrations. Analyses were performed for each mouse strain separately. Ester (a) and ether (e) bonds linking the fatty acids to the glycerol moiety are specified in the metabolite labels: acyl (a), diacyl (aa), acyl alkyl (ae).

To investigate changes in the plasma metabolome during the development of young mice from adolescence to young adulthood, metabolomics data were evaluated individually for each mouse strain. Since metabolite levels are known to be sex dependent, male and female mice were analyzed separately. Principle component analysis (PCA) revealed defined but overlapping clusters between 6 and 20 weeks of age in all four mouse strains (Figure 1A). This observation indicates a fairly smooth transition of the metabolite profiles during murine development. However, in male mice the clusters for six weeks of age were clearly separated, indicating the presence of distinct differences in metabolite profiles. In contrast, the clusters for 10, 14, and 20 weeks of age overlapped to different extents. In comparison to male mice, clusters of female mice generally overlapped more with only B6NTac and C3Fe mice, showing distinct clusters at 20 weeks of age.

Identification of the metabolites that distinguished between the sampling time points yielded sex- and strain-specific metabolite sets, as can be seen in the heatmaps in Figure 1B. While in general the most frequently identified metabolites were phosphatidylcholines (PC) with long- and very-long-chain fatty acids, the metabolite species composition and concentration patterns differed markedly between the strains and sexes. Female 129S1 mice showed high PC, lysophosphatidylcholine (lysoPC), and methionine levels at weeks 14 and 20 compared to weeks 6 and 10. Both male and female 129S1 mice showed higher levels of PC at 14 and 20 weeks. However, in males this was only true for PCs with acyl alkyl (ae) linkages. For PCs with diacyl (aa) linkages the reverse was true. Additionally, 129S1 males had high amino acid levels at weeks 6 and 20, compared to the levels at weeks 10 and 14. B6J females had particularly high amino acid levels at week 14, while PCs were found to be low at week 20. These mice also exhibited gradually decreasing levels of C16 acylcarnitine and hexoses. B6J males, however, had high levels of acylcarnitines at week six and high levels of phenylalanine, ornithine, and tyrosine at week 14. Like B6J, B6NTac females had low PC levels only at an age of 20 weeks. In contrast, lysoPC concentrations were increased at this age. Male B6NTac mice differed from their female counterparts with PC levels being particularly low at week six, while at that age sphingomyelin (SM) levels were increased. C3Fe mice stood out because female and male mice had a high similarity in identified age-dependent PC species and concentration patterns. Female C3Fe mice showed high levels of glutamine and histidine at 6 and 14 weeks of age. Ornithine levels were increased in males at 14 and 20 weeks.

### 2.2. Metabolomic Similarities during Adolescence and Early Adulthood

**Figure 2:**
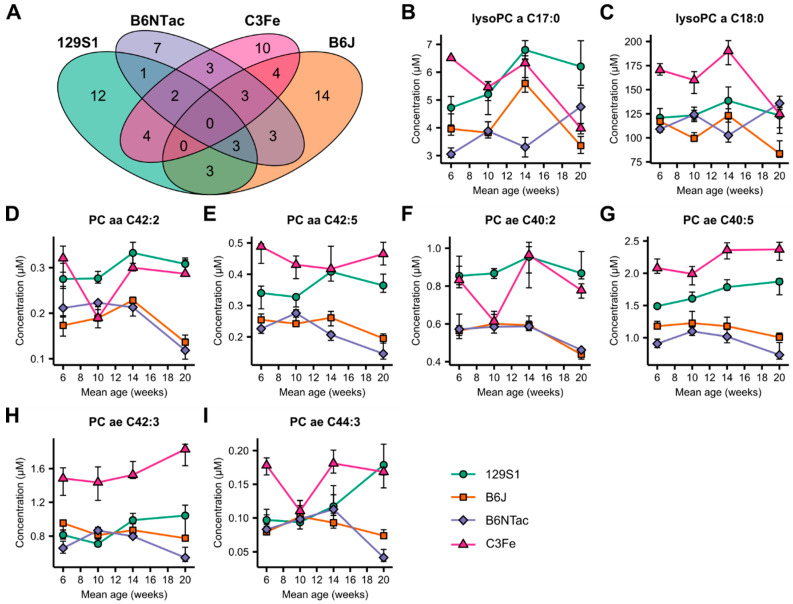
Inter-strain comparison of age-dependent changes in the plasma metabolome of female mice. (**A**) Venn diagram depicting the overlap of strain-specific age-dependent metabolites. (**B**–**I**) Line-dot plots of metabolites showing age-dependent changes in three out of four mouse strains. Dots and error bars represent median and interquartile range (IQR), respectively. Fold changes and *p*-values can be found in Supplementary Table S5.

**Figure 3:**
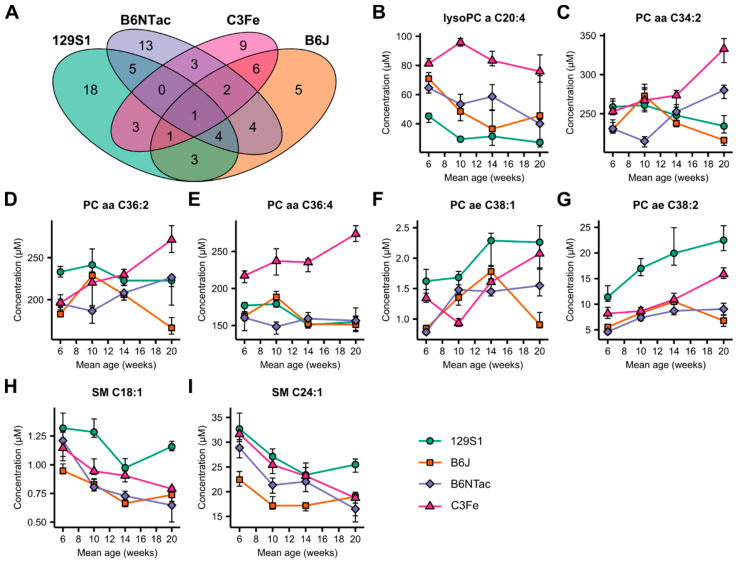
Inter-strain comparison of age-dependent changes in the plasma metabolome of male mice. (**A**) Venn diagram depicting the overlap of strain-specific age-dependent metabolites. (**B**–**I**) Line-dot plots of metabolites showing age-dependent changes in at least three out of four mouse strains. PC ae C38:2 (**G**) detection was age dependent in all four mouse strains. Dots and error bars represent median and IQR, respectively. Fold changes and *p*-values can be found in Supplementary Table S6.

Having investigated the strain-specific metabolomic changes in developing mice, we were interested in identifying age-related metabolites that were common in all mouse strains. We analyzed the overlap among the strain-specific age-related metabolite sets separately for each sex (Figure 2A and Figure 3A). While in female mice no common metabolite was found across all strains, one metabolite was identified in males: PC ae C38:2.

We then relaxed the stringency of our analysis and searched for metabolites that overlapped in at least three out of four strains (Figure 2B–I and Figure 3B–I). Under these conditions, eight metabolites were identified in females, consisting of two lysoPCs and six PCs (Figure 2B–I, Supplementary Table S5). Although all metabolites were identified as age-related, the actual metabolite levels and patterns of concentration changes differed markedly between strains. Only B6J and B6NTac mice had similarities in PC levels at all sampled time points. In male mice, a set of eight metabolites was also identified, comprising one lysoPC, five PCs, and two sphingomyelins (Figure 3B–I, Supplementary Table S6). LysoPC, a C20:4, had a general downward trend with increasing age, but at different concentration levels for each strain. In contrast, PC levels followed no general pattern in the different strains. Only male C3Fe mice exhibited increasing concentrations in all five PC species. Male B6J and B6NTac mice were less similar in their PC levels than were female mice. The sphingomyelins SM C18:1 and SM C24:1 in male mice showed a general downward trend in all strains. However, 129S1 and B6J mice exhibited increased levels of these sphingomyelins at week 20.

In summary, we found no age-related, strain-spanning set of metabolites that would suggest the presence of general, conserved underlying metabolic processes for mice developing from adolescents to young adults. Little overlap was found in the age-related plasma metabolome of the mouse strains tested. The concentration courses of the identified metabolites differed markedly between the strains. Similarities were only observed in B6J and B6NTac mice.

### 2.3. Metabolomic Differences in Age-Matched Mouse Strains

**Figure 4:**
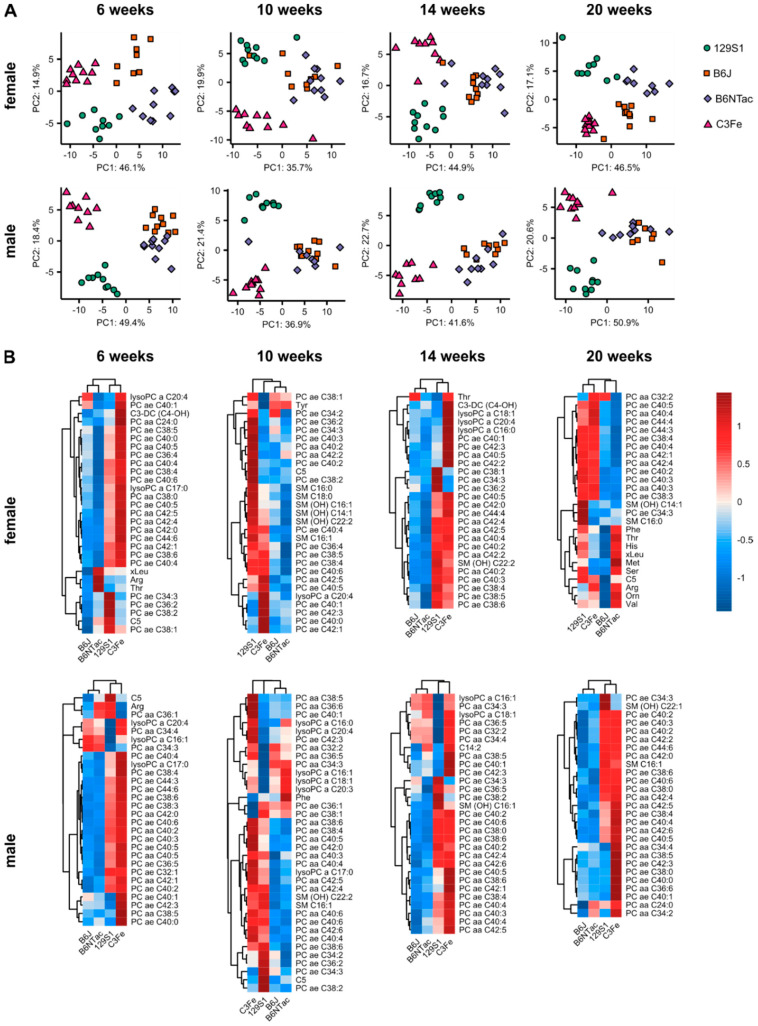
Differences in the plasma metabolome of age-matched mouse strains. (**A**) PCA of metabolomics data at the time points 6, 10, 14, and 20 weeks of age. (**B**) Heatmaps of z scores of top metabolites differentiating between strains at the different time points. Metabolites were selected based on loading scores of principle components 1 to 3 in PCA and the mean decrease in accuracy in random forest analysis. Z scores were calculated from scaled and centered mean metabolite concentrations.

Having seen the extent of the differences between the metabolomes of different mouse strains during adolescence and early adulthood, we looked for metabolite patterns that clearly distinguished the strains (Figure 4A). At six weeks of age, PCA clearly revealed distinct clusters for every mouse strain in both males and females. However, the clustering changed with advancing age. At 10 and 14 weeks of age, the metabolite levels of B6J and B6NTac mice became so similar that a clear distinction between the two was not possible. At the age of 20 weeks, females of both strains became distinguishable again, while male mice retained a strong overlap. The clusters of 129S1 and C3Fe mice remained separated throughout the study. The variance of metabolite profiles within the groups was found to be dependent on the strain, sex, and age of the mice. Females generally had higher heterogeneity in the scattering of the clusters than their male counterparts. While female C3Fe mice showed concise metabolite profiles at ages of 6 and 20 weeks, their clusters were spread widely at 10 and 14 weeks.

Depending on age, different sets of metabolites distinguished the strains, as shown in the heatmaps in Figure 4B. In general, PCs with long- or very-long-chain fatty acids were the major differentiators at every time point. 129S1 and C3Fe, for example, had higher PC concentrations than B6J and B6NTac mice. However, the individual species among the discriminating PCs changed with age. Besides phosphatidylcholines, metabolites of other classes were found to differentiate between the examined mouse strains. High levels of C5-carnitine and different sphingomyelins were detected in 129S1 mice at multiple time points but were not observed in the other strains. 129S1 mice had the highest levels of C5-carnitine throughout the study, but the extent of differentiation was sex and age dependent (Supplementary Figure S1). Amino acids like arginine and threonine were found to distinguish between the different strains predominantly in female mice. Higher amino acid levels were also found in female B6NTac and 129S1 mice at 20 weeks of age than in B6J and C3Fe mice. In conclusion, the mouse strains examined were found to have specific metabolite profiles that differentiated them from each other, while the extent of differentiation depended on the sex and age of the animals.

## 3. Discussion

### 3.1. Metabolomic Changes during Adolescence and Early Adulthood

Young laboratory mice aged between 6 and 20 weeks are commonly used in animal experiments, including in metabolomics research [14]. However, mice at such a young age undergo comprehensive physiological changes, which might influence experimental outcomes [11,12,13]. Our results clearly show age-related changes in the plasma metabolome of females and males of four mouse strains between 6 and 20 weeks of age (Figure 1A). Between 6 and 10 weeks of age, male mice showed the most extensive metabolomic differences. In contrast, females had highly similar metabolite profiles at both time points, with the sole exception of C3Fe mice. The age range 6 to 10 weeks covers parts of adolescence and puberty, which are intimately intertwined but distinguishable development phases. Adolescence is defined as the period in which development of adult cognitive and social behavior occurs. It starts after weaning at approximately three weeks and continues until the beginning of adulthood at nine weeks of age. Puberty refers to the attainment of gonadal and sexual maturity. Mature spermatozoa are present in male mice around five weeks of age [15]. In females, vaginal opening occurs at around four weeks, and first estrus occurs between 5 and 12 weeks [16,17,18,19,20]. However, the exact age at which puberty is attained varies depending on mouse strain and housing conditions, as well as the indicator examined. During adolescence, mice exhibit increased food consumption and energy expenditure [21]. Neurobiological remodeling leads to behavioral changes like increased locomotor activity and risk-taking behavior [22,23,24]. Increasing levels of gonadotropins and sex steroids play a role in these changes but also facilitate sexual maturation and the development of secondary sex characteristics [22,25]. Since female mice of most strains were found to have only minor metabolomic differences between 6 and 10 weeks of age, we assume that the majority of changes associated with adolescence and puberty happened before the age of six weeks. In contrast, male mice had pronounced metabolomic differences between the two time points. This observation supports the assumption that at least part of adolescent and pubertal developmental took place between 6 and 10 weeks of age. The differences between C3Fe females and female mice of the other strains showed that developmental timing is not only sex but also strain specific. Nelson et al. found significant differences in the timing of female pubertal development between C3H/HeJ mice, which have a common origin with C3Fe, and B6J mice [19].

Limited research has been done into physiological changes occurring between 10 and 20 weeks of age. Mice reach adulthood at nine weeks of age but are usually considered mature adults at an age of three to six months, when they are past development but not yet senescent. The first senescent changes are not observed before 10 months of age, and age-related biomarkers are detectable at 18 months [11]. However, development and aging are a continuum, and physiological changes do not pause during adulthood.

Somerville et al. showed that body mass, bone mineralization, and tibial length continued to increase between three and six months of age in C57BL/6 mice [12]. We also observed significant body weight gains between 10 and 20 weeks of age in most mouse strains (Supplementary Figure S2). Pinchuk et al. found age- and strain-dependent differences in T cell and antigen presenting cell populations in three and five month old BALB/c and C57BL/6 mice [13]. Body composition, bone mineral density and immune status are all known to influence the metabolome [26,27,28]. We discovered the metabolomic changes between 10 and 20 weeks of age to be highly strain specific in both sexes. While the metabolite profiles of B6NTac and C3Fe mice were highly similar between 10 and 14 weeks of age, they were clearly distinct in 129S1 mice. B6J mice were found to be intermediate in similarity. Mouse strains with few metabolomic differences between 10 and 14 weeks of age, usually had extensive changes between 14 and 20 weeks of age, and vice versa. Our results clearly demonstrate the variability of the plasma metabolome during adolescence and in early adulthood.

While we observed high similarities in metabolite profiles between consecutive time points, these timeframes were sex and strain specific. It is uncertain whether temporary fluctuations happened between time points that appeared to be stable. Due to the limits of the observed age range and the temporal resolution of measurements, it remains unclear whether the murine metabolome reaches a stable minimum at some point in life. Consideration should also be given to the possibility of metabolic phenotypes being enhanced or masked due to metabolomic variability. The analysis of metabolome and disease-relevant changes in a knockout mouse model of a metabolic disease covering postnatal development with a high temporal resolution could help answer these questions. However, our results are solely based on the plasma levels of lipid species and amino acids covered by the assay. It thereby sheds light on one section of the metabolomic changes occurring during murine postnatal development. Depending on the experimental setup as well as the metabolites and tissues of interest, the observed variability may differ.

Until these uncertainties are resolved, it is of utmost importance to minimize age differences not only within experiments but also in subsequent experiments. This consistency would keep sample variance low and allow the establishment of connections between the results of experiments that build upon one another. Animal age also needs to be reported precisely to ensure the reproducibility and comparability of study outcomes.

### 3.2. Strain-Specific Metabolomes during Adolescence and Early Adulthood

After seeing the way in which the metabolite profiles of mouse strains evolved with age, we were interested in similarities that could suggest general mechanisms of development. However, we only found eight sex-specific metabolites in females and males that were considered age dependent in at least three out of four mouse strains. Within this selection, only a few metabolites had similar levels and concentration courses between the strains (Figure 2 and Figure 3). PCA revealed distinct clusters for each mouse strain, indicating the existence of specific metabolite profiles at six weeks of age. While 129S1 and C3Fe clusters remained distinctive, B6J and B6NTac became highly similar at a later age. Females became distinguishable again at 20 weeks of age, while male mice remained highly similar (Figure 4).

The reasons for these findings lie in the genetics of the mice. Genomic and proteomic differences among inbred mouse strains are well known and translate into strain-specific phenotypes and metabolomes [29,30,31,32,33,34]. Genetic diversity between mouse strains is mainly found at loci related to immunity but also at those involved in sexual reproduction [29]. The fact that the immune system and sexual maturation are still under development at the age of 6 to 20 weeks might explain the observed metabolomic changes [11,13]. Part of these genetically induced metabolomic differences may also arise due to an offset of the developmental clock. The onset of puberty is known to depend on genetic factors as well as body composition or energy balance [16,17,22]. While body composition was not analyzed in this study, strain-specific differences in body weight development were observed (Table 1, Supplementary Figure S2). Differences in timing of development might also explain the alternating similarities of B6J and B6NTac mice. These inbred strains were separated in 1951 and were shown to be genetically and phenotypically distinct [35,36]. In this study they also showed differences in body weight development. Metabolomics analysis, however, indicates that B6J and B6NTac mice share a common origin (Figure 4).

Genetic differences can also lead to specific metabolic abnormalities. Mice of strain 129S1 had high levels of C5-carnitine throughout the study, an observation that has previously been reported (Figure 4B). In the related substrain 129S2/SvPasCrl, Leandro et al. showed that a mutation in the *Ivd* gene, encoding the isovaleryl-CoA dehydrogenase, is responsible for the accumulation of C5-carnitine and thereby mimics the metabolic disorder isovaleric acidemia [32]. Fujisaka et al. reported four times higher C5-carnitine levels in male 129S1 mice compared to B6J mice at 11 weeks of age [33]. We found significant fluctuations in C5-carnitine levels (Supplementary Figure S1). Compared with B6J, 129S1 females had C5-carnitine levels that were only 1.6 times higher at 14 weeks, but five times higher at 6 weeks of age. Male 129S1 mice showed levels between 2.7 times and 5.2 times higher than their B6J counterparts. While C5-carnitine concentrations remained significantly increased in 129S1 at all points in time, the fluctuations observed indicate that study outcomes are prone to the influence of age.

Our findings indicate that metabolomics studies that use mice with different genetic backgrounds are not easily comparable. Genetic differences lead to different metabolite profiles that develop differently with age. Caution is especially advised when comparing results of genetically similar substrains. While metabolite profiles might be comparable at a certain age, they can be inherently different a few weeks earlier or later.

## 4. Materials and Methods

### 4.1. Mice

For this study, C3Fe, B6J, B6NTac, and 129S1 mice were used. C3Fe and B6J strains were imported from the Jackson Laboratory (Bar Harbor, ME, USA) and bred in the animal facilities of the Helmholtz Zentrum München. B6NTac mice were imported from Taconic Biosciences (Leverkusen, Germany), while 129S1 mice were imported from Charles River (Sulzfeld, Germany). All animal experiments were approved by the District Government of Upper Bavaria and the national ethics committee (approval code 70/07). A group size of 10 mice per strain and sex was chosen. Animals that died during the course of the study were replaced, if possible, with mice of a similar age. A total of 82 mice was used in this study. The mice were housed in the animal facility of the German Mouse Clinic (Helmholtz Zentrum, München, Germany) in individually ventilated caging systems under specific pathogen-free conditions. They had *ad libitum* access to sterile-filtered tap water in bottles, and an irradiated breeding diet (1314, Altromin, Lage, Germany). Room temperature and relative humidity were kept at 22 ± 2 °C and 55 ± 10%, respectively. The lighting was set to a 12-h light/dark cycle, with lights on at 6 a.m. The health status of the animals was checked on a weekly basis.

### 4.2. Plasma Sample Collection and Preparation

Mouse plasma samples were collected at mean ages of 6, 10, 14, and 20 weeks. The group size ranged from 8 to 10 mice per sex, strain, and sampling time point. Before blood collection, the mice were weighed. Blood sampling was conducted under isoflurane anesthesia by retro-orbital venous sinus puncture, using non-heparinized glass capillaries with a diameter of 0.8 mm. The blood was collected into EDTA-coated tubes (1000 A, KABE Labortechnik, Nümbrecht-Elsenroth, Germany). Blood samples were centrifuged for 10 min at 2000× *g* and 4 °C within 45 min after sampling. Plasma supernatants were transferred into fresh collection tubes, immediately snap frozen using dry ice and stored at −80 °C until use.

### 4.3. Targeted Metabolomics

Metabolites were quantified using Absolute*IDQ*™ p150 Kits (BIOCRATES Life Sciences AG, Innsbruck, Austria). The assay uses flow injection-electrospray ionization-tandem mass spectrometry (FIA-ESI-MS/MS) for the quantification of up to 163 metabolites. It covers six different metabolite classes, including 41 acylcarnitines (Cx:y), 14 amino acids, hexoses (sum of hexoses, approx. 90–95% glucose), 15 lysophosphatidylcholines (lysoPC), 77 phosphatidylcholines (PC), and 15 sphingomyelins (SMx:y). In these generic formulae, the placeholders x and y represent the total number of carbons and double bonds of all chains, respectively.

The assay procedure followed the manufacturer’s instructions, and has been described previously in great detail [37]. Sample preparation was conducted with the help of a Microlab STAR™ liquid handling system (Hamilton Bonaduz AG, Bonaduz, Switzerland) and an Ultravap^®^ nitrogen evaporator (Porvair Sciences, Leatherhead, UK). Mass spectrometry was performed with a 4000 QTrap™ triple quadrupole mass spectrometer (Sciex Deutschland GmbH, Darmstadt, Germany) equipped with a Shimadzu Prominence HPLC (Shimadzu Deutschland GmbH, Duisburg, Germany) and controlled by Analyst software 1.5 (Sciex Deutschland GmbH, Darmstadt, Germany). Data analysis for metabolite quantification and quality assessment was performed using Met*IDQ*™ software 6.4.8, which is part of the Absolute*IDQ*™ Kit. Metabolite concentrations were calculated using internal standards and reported in µM. The assay’s metabolite-specific limits of detection (LOD) have been determined experimentally by Biocrates.

### 4.4. Statistical Analysis

Data processing and analysis were performed using the R software environment 3.6.1 [38]. Metabolites with more than 80% of samples below LOD were removed from the dataset. Out of 163 metabolites quantified with the assay, 120 metabolites fulfilled this criterion. To facilitate statistical analysis, metabolite concentrations equaling zero were imputed by multiplication of the respective zero sample (PBS) concentration with a random factor between 0.75 and 1.25. If the zero sample value was also zero, the minimal sample concentration measured among all samples of the same kit plate was used for the imputation instead. To account for batch effects, the dataset was normalized using Biocrates QC samples that were measured on every kit plate. Metabolite concentrations were log_2_-transformed, and outliers were identified using the interquartile range (IQR) as well as the first quartile (Q_1_) and third quartile (Q_3_). Outliers were defined as metabolite concentrations bigger than Q_3_ + 1.5 × IQR or smaller than Q_1_ − 1.5 × IQR. Samples with more than 25% outliers were removed from the dataset. The remaining samples were used for further statistical analysis. The corresponding number of animals per group, as well as the mean body weight and age, are specified in Table 1.

PCA was performed for data exploration and metabolite selection using scaled and centered metabolite concentrations. The first three principal components were selected for further analysis. Loading scores of metabolites highly correlated with the principle components 1, 2, and 3 can be found in the Supplementary Tables S1 to S4. Random forest analysis, a supervised machine learning method, was also used for metabolite selection. The number of variables randomly sampled for each split was set to the square root of the number of metabolites analyzed. The number of decision trees was set to 1000. Metabolites were selected for further analysis when they met the conditions to be among the top 20 metabolites with the highest loading scores of principal components 1, 2 or 3 in the PCA, as well as among the top 50 metabolites with the highest mean decrease in accuracy using random forest analysis. Two-sided Student’s *t*-tests were performed to determine statistical significance in the body weight data. Two-sided Wilcoxon rank sum tests were performed to determine statistical significance in the metabolomics data. Received *p*-values were corrected for multiple testing using the Benjamini and Hochberg procedure [39].

## 5. Conclusions

In conclusion, we showed that the murine plasma metabolome is not only strain specific but also highly variable within the analyzed age range of 6 to 20 weeks, a range upon which most studies involving mice rely. Unfortunately, the identification of a universal age range with minimal metabolomic variability remains elusive. While strain-specific age ranges with highly similar metabolite profiles were detected, the presence of temporal fluctuations in metabolite levels in the intervals between sampling time points cannot be ruled out. Further studies covering a larger timeframe with higher temporal resolution should be carried out to address these issues. Apart from the necessity of making deliberate strain choices, we feel compelled to emphasize the importance of consistency and precise documentation of animal age in any study, but especially in metabolome research.

## Figures and Tables

**Table 1:** Animal data. Mean age in weeks at blood sampling calculated from the entire cohort.

	Mean Age	129S1		B6J		B6NTac		C3Fe	
	(Weeks)	Female	Male	Female	Male	Female	Male	Female	Male
*n*		9	10	8	10	10	10	10	9
body weight (g)	6	21.2 ± 1.4	22.5 ± 1.4	17.3 ± 1.0	21.8 ± 0.9	17.6 ± 1.2	19.8 ± 1.3	21.2 ± 1.5	24.1 ± 1.4
age (d)		42 ± 0	42 ± 0	46 ± 1	46 ± 0	42 ± 0	42 ± 0	44 ± 2	45 ± 3
*n*		10	9	9	10	10	8	10	10
body weight (g)	10	25.2 ± 3.5	27.5 ± 2.0	19.5 ± 0.8	26.4 ± 1.2	20.2 ± 1.3	25.1 ± 2.0	24.3 ± 1.6	28.0 ± 1.4
age (d)		70 ± 0	70 ± 0	74 ± 0	74 ± 0	70 ± 0	70 ± 0	72 ± 2	73 ± 3
weight gain (g/d)		0.14	0.18	0.08	0.16	0.10	0.19	0.11	0.14
*n*		10	10	9	9	10	9	9	9
body weight (g)	14	26.9 ± 5.2	30.2 ± 2.2	21.0 ± 1.0	28.6 ± 1.1	21.8 ± 2.0	28.4 ± 2.3	27.2 ± 2.0	31.1 ± 1.5
age (d)		98 ± 0	98 ± 0	106 ± 4	104 ± 0	99 ± 0	98 ± 0	100 ± 2	101 ± 3
weight gain (g/d)		0.06	0.09	0.05	0.07	0.06	0.12	0.10	0.11
*n*		9	10	10	8	8	9	10	10
body weight (g)	20	28.9 ± 6.3	32.5 ± 3.1	21.7 ± 0.9	29.9 ± 1.9	25.2 ± 3.4	32.4 ± 2.8	33.0 ± 2.6	36.1 ± 2.2
age (d)		133 ± 0	133 ± 0	147 ± 4	145 ± 0	140 ± 0	140 ± 0	151 ± 2	152 ± 3
weight gain (g/d)		0.06	0.07	0.02	0.03	0.08	0.09	0.12	0.10

The live animal body weight in grams (g) and age in days (d) are given as mean ± SD. The weight gain in grams per day (g/d) depicts the increase in body weight since the last blood sampling per days past. *n*: number of animals per group.

## Data Availability

The datasets generated and analyzed during the current study are available from the corresponding author on reasonable request.

## Supplementary Materials

The following are available online at https://www.mdpi.com/2218-1989/10/11/472/s1. Figure S1: Age-dependent changes of C5-carnitine levels in female and male mice, Figure S2: Body weight development of different mouse strains, Table S1: Selected PCA loading scores for age-dependent metabolomic changes in female mice, Table S2: Selected PCA loading scores for age-dependent metabolomic changes in male mice, Table S3: Selected PCA loading scores for females of age-matched mouse strains, Table S4: Selected PCA loading scores for males of age-matched mouse strains. Table S5: Fold changes of selected metabolites showing overlap in age regulation among female mice of different strains, Table S6: Fold changes of selected metabolites showing overlap in age regulation among male mice of different strains.

## References

[1] Wishart D.S. (2016). Emerging applications of metabolomics in drug discovery and precision medicine. Nat. Rev. Drug Discov..

[2] Bujak R., Struck-Lewicka W., Markuszewski M.J., Kaliszan R. (2015). Metabolomics for laboratory diagnostics. J. Pharm. Biomed. Anal..

[3] (2020). 2019 Report on the Statistics on the Use of Animals for Scientific Purposes in the Member States of the European Union in 2015–2017.

[4] Dyar K.A., Lutter D., Artati A., Ceglia N.J., Liu Y., Armenta D., Jastroch M., Schneider S., de Mateo S., Cervantes M. (2018). Atlas of Circadian Metabolism Reveals System-wide Coordination and Communication between Clocks. Cell.

[5] Bassett S.A., Young W., Fraser K., Dalziel J.E., Webster J., Ryan L., Fitzgerald P., Stanton C., Dinan T.G., Cryan J.F. (2019). Metabolome and microbiome profiling of a stress-sensitive rat model of gut-brain axis dysfunction. Sci. Rep..

[6] Jensen T.L., Kiersgaard M.K., Sørensen D.B., Mikkelsen L.F. (2013). Fasting of mice: A review. Lab. Anim..

[7] Overmyer K.A., Thonusin C., Qi N.R., Burant C.F., Evans C.R. (2015). Impact of anesthesia and euthanasia on metabolomics of mammalian tissues: Studies in a C57BL/6J mouse model. PLoS ONE.

[8] Houtkooper R.H., Argmann C., Houten S.M., Cantó C., Jeninga E.H., Andreux Ṕ.A., Thomas C., Doenlen R., Schoonjans K., Auwerx J. (2011). The metabolic footprint of aging in mice. Sci. Rep..

[9] Tomás-Loba A., Bernardes de Jesus B., Mato J.M., Blasco M.A. (2013). A metabolic signature predicts biological age in mice. Aging Cell.

[10] Kim S., Cheon H.S., Song J.C., Yun S.M., Park S.I., Jeon J.P. (2014). Aging-related Changes in Mouse Serum Glycerophospholipid Profiles. Osong Public Health Res. Perspect..

[11] Dutta S., Sengupta P. (2016). Men and mice: Relating their ages. Life Sci..

[12] Somerville J.M., Aspden R.M., Armour K.E., Armour K.J., Reid D.M. (2004). Growth of C57Bl/6 Mice and the Material and Mechanical Properties of Cortical Bone from the Tibia. Calcif. Tissue Int..

[13] Pinchuk L.M., Filipov N.M. (2008). Differential effects of age on circulating and splenic leukocyte populations in C57BL/6 and BALB/c male mice. Immun. Ageing.

[14] Jackson S.J., Andrews N., Ball D., Bellantuono I., Gray J., Hachoumi L., Holmes A., Latcham J., Petrie A., Potter P. (2017). Does age matter? The impact of rodent age on study outcomes. Lab. Anim..

[15] Brust V., Schindler P.M., Lewejohann L. (2015). Lifetime development of behavioural phenotype in the house mouse (Mus musculus). Front. Zool..

[16] Qiu X., Dowling A.R., Marino J.S., Faulkner L.D., Bryant B., Brüning J.C., Elias C.F., Hill J.W. (2013). Delayed Puberty but Normal Fertility in Mice With Selective Deletion of Insulin Receptors From Kiss1 Cells. Endocrinology.

[17] Chehab F.F., Mounzih K., Lu R., Lim M.E. (1997). Early Onset of Reproductive Function in Normal Female Mice Treated with Leptin. Science.

[18] Keri R.A., Lozada K.L., Abdul-Karim F.W., Nadeau J.H., Nilson J.H. (2000). Luteinizing hormone induction of ovarian tumors: Oligogenic differences between mouse strains dictates tumor disposition. Proc. Natl. Acad. Sci. USA.

[19] Nelson J.F., Karelus K., Felicio L.S., Johnson T.E. (1990). Genetic Influences on the Timing of Puberty in Mice. Biol. Reprod..

[20] Krewson T.D., Supelak P.J., Hill A.E., Singer J.B., Lander E.S., Nadeau J.H., Palmert M.R. (2004). Chromosomes 6 and 13 Harbor Genes that Regulate Pubertal Timing in Mouse Chromosome Substitution Strains. Endocrinology.

[21] Moore E.M., Linsenbardt D.N., Melón L.C., Boehm S.L. (2011). Ontogenetic differences in adolescent and adult C57BL/6J and DBA/2J mice: Anxiety-like, locomotor, and consummatory behaviors. Dev. Psychobiol..

[22] Sisk C.L., Foster D.L. (2004). The neural basis of puberty and adolescence. Nat. Neurosci..

[23] Romeo R.D., Richardson H.N., Sisk C.L. (2002). Puberty and the maturation of the male brain and sexual behavior: Recasting a behavioral potential. Neurosci. Biobehav. Rev..

[24] Laviola G., Macrì S., Morley-Fletcher S., Adriani W. (2003). Risk-taking behavior in adolescent mice: Psychobiological determinants and early epigenetic influence. Neurosci. Biobehav. Rev..

[25] Bell M.R. (2018). Comparing Postnatal Development of Gonadal Hormones and Associated Social Behaviors in Rats, Mice, and Humans. Endocrinology.

[26] Zhao H., Shen J., Djukovic D., Daniel-MacDougall C., Gu H., Wu X., Chow W.H. (2016). Metabolomics-identified metabolites associated with body mass index and prospective weight gain among Mexican American women. Obes. Sci. Pract..

[27] Chuang T.-L., Lin J.-W., Wang Y.-F. (2019). Bone Mineral Density as a Predictor of Atherogenic Indexes of Cardiovascular Disease, Especially in Nonobese Adults. Dis. Markers.

[28] de Goede K.E., Harber K.J., Van den Bossche J. (2019). Let’s Enter the Wonderful World of Immunometabolites. Trends Endocrinol. Metab..

[29] Lilue J., Doran A.G., Fiddes I.T., Abrudan M., Armstrong J., Bennett R., Chow W., Collins J., Collins S., Czechanski A. (2018). Sixteen diverse laboratory mouse reference genomes define strain-specific haplotypes and novel functional loci. Nat. Genet..

[30] Timmermans S., Van Montagu M., Libert C. (2017). Complete overview of protein-inactivating sequence variations in 36 sequenced mouse inbred strains. Proc. Natl. Acad. Sci. USA.

[31] Michaud S.A., Sinclair N.J., Pětrošová H., Palmer A.L., Pistawka A.J., Zhang S., Hardie D.B., Mohammed Y., Eshghi A., Richard V.R. (2018). Molecular phenotyping of laboratory mouse strains using 500 multiple reaction monitoring mass spectrometry plasma assays. Commun. Biol..

[32] Leandro J., Violante S., Argmann C.A., Hagen J., Dodatko T., Bender A., Zhang W., Williams E.G., Bachmann A.M., Auwerx J. (2019). Mild inborn errors of metabolism in commonly used inbred mouse strains. Mol. Genet. Metab..

[33] Fujisaka S., Avila-Pacheco J., Soto M., Kostic A., Dreyfuss J.M., Pan H., Ussar S., Altindis E., Li N., Bry L. (2018). Diet, Genetics, and the Gut Microbiome Drive Dynamic Changes in Plasma Metabolites. Cell Rep..

[34] Qiao Q., Li T., Sun J., Liu X., Ren J., Fei J. (2011). Metabolomic analysis of normal (C57BL/6J, 129S1/SvImJ) mice by gas chromatography–mass spectrometry: Detection of strain and gender differences. Talanta.

[35] Mekada K., Abe K., Murakami A., Nakamura S., Nakata H., Moriwaki K., Obata Y., Yoshiki A. (2009). Genetic differences among C57BL/6 substrains. Exp. Anim..

[36] Zurita E., Chagoyen M., Cantero M., Alonso R., González-Neira A., López-Jiménez A., López-Moreno J.A., Landel C.P., Benítez J., Pazos F. (2011). Genetic polymorphisms among C57BL/6 mouse inbred strains. Transgenic Res..

[37] Römisch-Margl W., Prehn C., Bogumil R., Röhring C., Suhre K., Adamski J. (2012). Procedure for tissue sample preparation and metabolite extraction for high-throughput targeted metabolomics. Metabolomics.

[38] R Core Team (2020). R A Language and Environment for Statistical Computing.

[39] Benjamini Y., Hochberg Y. (1995). Controlling the False Discovery Rate: A Practical and Powerful Approach to Multiple Testing. J. R. Stat. Soc. Ser. B.

