# Supplementary material for: Mouse Age Matters: How Age Affects the Murine Plasma Metabolome
Source: Metabolites. 2020 Nov 19;10(11):472. doi: 10.3390/metabo10110472 (PMC7699431; doi:10.3390/metabo10110472)
Supplement: Supplementary file 1 [file metabolites-10-00472-s001.pdf]

Supplementary Materials for "Mouse Age Matters: How Age Affects the Murine Plasma Metabolome"

Patrick Pann [1], Martin Hrabě de Angelis [2,3,4], Cornelia Prehn [1] and Jerzy Adamski [1,3,4,5,*]

[1] Research Unit Molecular Endocrinology and Metabolism, Helmholtz Zentrum München, German Research Center for Environmental Health (GmbH), 85764 Neuherberg, Germany; patrick.pann@helmholtz-muenchen.de (P.P.); prehn@helmholtz-muenchen.de (C.P.)
[2] German Mouse Clinic, Institute of Experimental Genetics, Helmholtz Zentrum München, German Research Center for Environmental Health (GmbH), 85764 Neuherberg, Germany; hrabe@helmholtz-muenchen.de
[3] German Center for Diabetes Research (DZD), 85764 Neuherberg, Germany
[4] Chair of Experimental Genetics, School of Life Science, Weihenstephan, Technische Universität München, 85354 Freising, Germany
[5] Department of Biochemistry, Yong Loo Lin School of Medicine, National University of Singapore, Singapore 117597, Singapore
* Correspondence: adamski@helmholtz-muenchen.de

Supplementary Figures

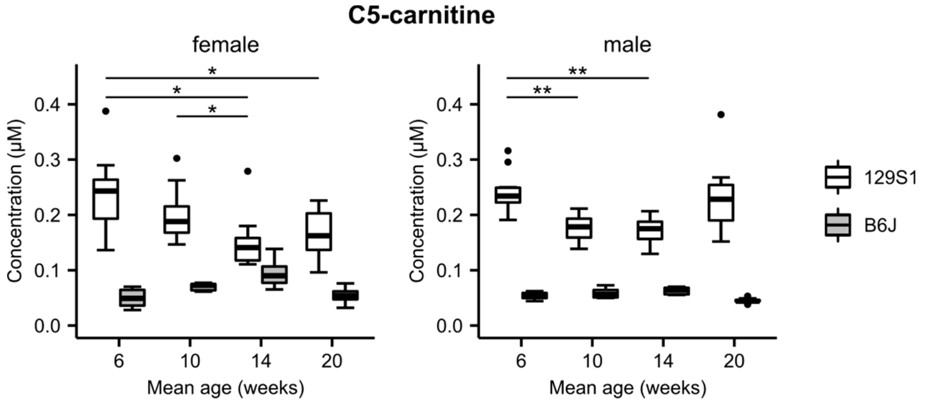

Supplementary Figure S1. Age-dependent changes of C5-carnitine levels in female and male mice. Changes were tested for significance using Wilcoxon rank sum tests. P-values were corrected for multiple testing using the Benjamini & Hochberg procedure and are given in brackets. * p ≤ 0.05, ** p ≤ 0.01.

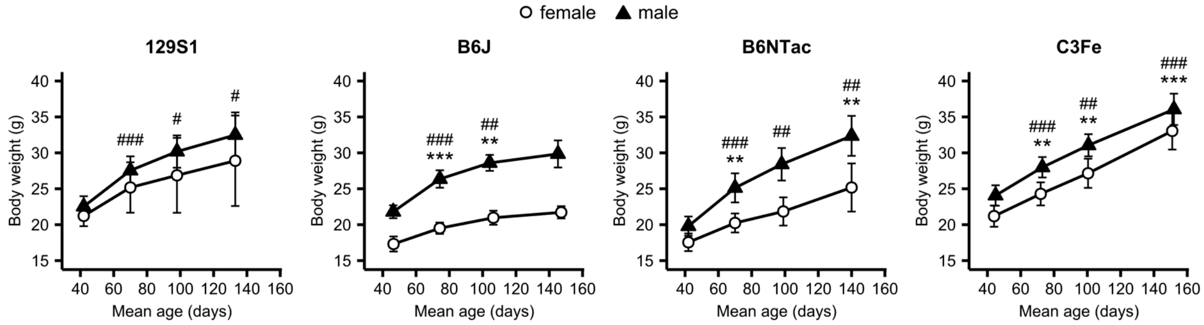

Supplementary Figure S2. Body weight development of different mouse strains. Animal body weight was measured at mean ages of 6, 10, 14, and 20 weeks directly before blood sampling. The exact age at sampling is given for each sex and strain as mean age in days. Data points for female and male mice are open circles and black triangles, respectively. Significance in body weight changes from 6 to 10,

10 to 14, and 14 to 20 weeks of age was calculated using Student's *t*-tests. P-values were corrected for multiple testing using the Benjamini & Hochberg procedure and are represented by * for females and # for males. */# $p \leq 0.05$, **/## $p \leq 0.01$. Data are expressed as mean ± SD.

Supplementary Tables

Supplementary Table S1. Selected PCA loading scores for age-dependent metabolomic changes in female mice. Metabolites with a correlation bigger than 0.85 with principle components 1, 2, or 3 for each individual mouse strain were selected. Loading scores with a correlation bigger than 0.85 are written in bold. Loading scores of metabolites selected for further analyses (see Figure 1B) are written in italics.

	129S1			B6J			B6NTac			C3Fe		
**Metabolite**	**PC1**	**PC2**	**PC3**	**PC1**	**PC2**	**PC3**	**PC1**	**PC2**	**PC3**	**PC1**	**PC2**	**PC3**
lysoPC a C20:4	-.069	-.061	.097	-.012	.018	-.214	**-.094**	.102	-.056	-.002	.022	-.253
PC aa C30:0	-.060	.071	-.073	-.075	.079	-.012	**-.138**	-.009	.029	-.030	.089	-.128
PC aa C32:1	-.010	-.060	-.227	-.049	.154	.144	**-.072**	-.015	.193	.151	.001	.018
PC aa C34:1	-.053	-.119	-.185	-.084	.127	.125	**-.069**	.002	.195	.140	.037	-.004
PC aa C34:4	-.092	-.090	.003	-.086	.155	.034	**-.133**	-.042	.042	.120	-.014	-.122
PC aa C36:1	-.045	-.121	-.177	-.126	.071	.041	**-.075**	.061	.154	.145	.028	-.023
PC aa C36:2	-.043	-.117	-.019	-.134	-.034	.054	**-.084**	.038	.113	.144	.007	-.058
PC aa C36:3	-.055	-.028	-.213	-.120	.018	.125	**-.079**	.021	.196	.150	-.005	-.046
PC aa C36:4	-.102	-.098	.046	-.114	.086	-.065	**-.132**	-.021	-.003	.103	-.019	-.189
PC aa C36:5	-.050	-.108	-.195	-.077	.158	.089	**-.077**	-.024	.183	.142	-.002	-.063
PC aa C38:0	-.138	.057	.058	-.132	-.052	-.045	**-.123**	-.006	-.099	-.012	.090	-.140
PC aa C38:3	-.085	-.125	-.130	-.096	.149	-.033	**-.061**	.060	.136	.139	.027	-.046
PC aa C38:5	-.119	-.043	-.109	-.061	.137	-.084	**-.122**	.004	.036	.144	-.014	-.095
PC aa C40:4	-.126	-.043	-.044	-.121	.101	.011	**-.116**	-.031	.045	.150	.035	-.030
PC aa C42:2	-.101	.074	-.015	-.134	-.011	.102	**-.127**	-.081	.038	.050	.143	.138
PC aa C42:4	-.130	.012	.053	-.133	.020	.012	**-.128**	-.069	-.027	.003	.150	-.034
PC aa C42:5	-.131	.032	.016	-.125	.081	.009	**-.133**	-.050	-.036	.071	.131	-.047
PC aa C42:6	-.114	-.080	-.069	-.066	.153	.087	**-.113**	-.093	.062	.145	-.011	-.085
PC ae C38:0	-.138	-.031	-.038	-.085	.157	.009	**-.129**	-.052	.017	.110	.034	-.097
PC ae C38:2	-.032	.165	.015	-.119	-.036	.117	**-.116**	-.004	.085	.151	.019	.023
PC ae C38:3	-.093	.127	-.103	-.125	-.041	.106	**-.120**	.014	.108	.141	.075	.059
PC ae C38:5	-.120	-.050	.024	-.135	.001	-.050	**-.128**	.049	-.038	.009	.089	-.159
PC ae C38:6	-.120	-.048	.087	-.139	-.013	-.037	**-.109**	.049	-.096	-.105	.107	-.104
PC ae C40:0	-.135	-.034	-.055	-.068	.037	-.092	**-.107**	-.015	-.030	.131	-.042	-.128
PC ae C40:1	-.134	.044	-.018	-.085	.123	-.097	**-.109**	.035	-.069	.082	.024	-.219
PC ae C40:2	-.128	.015	-.057	-.140	.004	.028	**-.126**	-.020	.079	.042	.156	.096
PC ae C40:3	-.118	.117	-.056	-.138	-.020	.050	**-.137**	.006	.042	.095	.120	.110
PC ae C40:4	-.097	.108	.121	-.133	-.040	-.045	**-.134**	.003	-.073	.107	.051	-.065
PC ae C40:5	-.141	.043	.002	-.133	-.012	-.057	**-.140**	.006	-.063	.114	.107	-.029
PC ae C42:1	-.133	.044	.026	-.106	.028	-.105	**-.133**	-.076	-.034	.050	.035	-.134
PC ae C42:2	-.108	.031	-.138	-.125	.087	.035	**-.136**	-.026	.056	.136	.075	.038
PC ae C42:3	-.133	.020	-.045	-.125	.073	-.055	**-.142**	-.029	-.007	.131	.060	-.064
SM C18:1	-.119	-.058	.061	-.041	-.031	-.217	**-.047**	.147	-.103	-.125	.099	-.062

Supplementary Table S2. Selected PCA loading scores for age-dependent metabolomic changes in male mice. Metabolites with a correlation bigger than 0.85 with principle components 1, 2, or 3 for each individual mouse strain were selected. Loading scores with a correlation bigger than 0.85 are written in bold. Loading scores of metabolites selected for further analyses (see Figure 1B) are written in italics.

	129S1			B6J			B6NTac			C3Fe		
**Metabolite**	**PC1**	**PC2**	**PC3**	**PC1**	**PC2**	**PC3**	**PC1**	**PC2**	**PC3**	**PC1**	**PC2**	**PC3**
C14	-.077	.118	-.045	-.029	-.160	.057	**-.051**	-.105	.195	-.054	.127	.003
C16	-.090	.123	-.106	-.021	-.172	.003	**-.042**	-.134	.182	-.085	.125	.062
C18:1	-.083	.109	-.143	-.012	-.174	.017	**-.043**	-.141	.128	-.059	.076	.152
C18:2	-.080	.100	-.140	-.035	-.166	-.014	**-.042**	-.148	.155	-.094	.089	.074
lysoPC a C16:0	.082	.072	.105	-.098	.001	.094	**-.129**	-.002	.062	-.028	.148	.031
lysoPC a C20:4	-.047	.165	-.022	.010	-.164	.102	**-.089**	-.145	.029	-.002	.040	.216
Orn	.070	-.001	.201	-.005	.165	.017	**-.040**	.113	-.026	.095	.038	-.145
PC aa C32:1	.043	.161	.075	-.124	.019	-.164	**-.046**	.129	-.036	.152	-.017	.000
PC aa C34:1	.032	.141	.026	-.140	.019	-.130	**-.086**	.068	-.061	.156	.001	-.021
PC aa C34:2	.008	.070	-.131	-.139	.043	-.084	**-.027**	.086	-.149	.149	-.033	-.016
PC aa C36:1	.088	.084	.015	-.147	.035	-.087	**-.116**	-.024	-.110	.159	.002	-.008
PC aa C36:2	.040	.067	-.111	-.140	.072	-.095	**-.076**	.064	-.169	.155	-.039	-.019
PC aa C36:3	.122	.057	-.010	-.151	.057	-.046	**-.099**	.122	-.064	.154	-.050	.003
PC aa C36:4	.015	.139	-.118	-.139	-.035	-.103	**-.124**	-.020	-.069	.149	-.039	.065
PC aa C38:3	.117	.101	.024	-.133	-.060	-.046	**-.116**	.034	-.019	.149	-.010	.048
PC aa C38:5	.007	.197	.005	-.052	-.148	.048	**-.117**	-.043	.026	.141	-.043	.103
PC aa C40:3	.150	-.049	-.029	-.142	.055	-.088	**-.068**	.180	.058	.143	.036	-.006
PC aa C40:4	.134	-.060	-.079	-.151	-.018	-.099	**-.087**	.147	.080	.155	-.027	.048
PC aa C40:5	-.021	.190	.002	-.074	-.149	-.043	**-.127**	-.005	.017	.117	-.003	.137
PC aa C42:5	.136	-.042	-.040	-.138	-.012	-.059	**-.085**	.143	.097	.147	.004	.050
PC ae C34:1	.105	.087	.005	-.142	.032	-.007	**-.129**	-.012	.024	.120	.080	.007
PC ae C36:3	.150	-.023	-.047	-.149	.061	-.059	**-.138**	.007	.015	.131	.026	.053
PC ae C36:4	.094	.007	-.104	-.131	-.005	.007	**-.130**	.001	.076	-.021	.108	.118
PC ae C36:5	.114	-.051	-.051	-.084	.095	.067	**-.133**	-.015	.008	.063	.097	.038
PC ae C38:1	.151	-.020	.047	-.091	.139	.045	**-.063**	.170	.010	.129	.053	-.116
PC ae C38:2	.137	-.102	-.004	-.088	.148	.005	**-.052**	.173	-.019	.149	-.025	-.071
PC ae C38:3	.159	-.012	.033	-.107	.107	.077	**-.107**	.119	.015	.146	.056	-.080
PC ae C38:4	.096	.016	-.132	-.067	-.081	.095	**-.129**	-.049	.027	.047	.012	.171
PC ae C38:5	.061	.098	-.113	-.137	-.030	.059	**-.135**	-.026	.069	.052	.074	.126
PC ae C38:6	.074	.007	-.126	-.150	.008	-.022	**-.110**	-.106	.026	.010	.127	.120
PC ae C40:1	.103	.116	-.045	-.072	-.131	.053	**-.120**	-.086	-.015	.068	-.027	.216
PC ae C40:2	.161	.035	-.030	-.138	.079	.030	**-.131**	.019	-.006	.066	.145	-.081
PC ae C40:3	.163	-.014	.017	-.118	.091	.073	**-.090**	.154	.055	.111	.099	-.106
PC ae C40:5	.097	.146	.002	-.134	-.023	.122	**-.135**	.001	.091	.081	.128	.017
PC ae C42:3	.104	.106	.046	-.114	-.058	.082	**-.133**	-.023	.012	.126	.042	.096
Phe	.086	.048	.187	.025	.157	.091	**-.089**	.040	-.123	.085	.086	-.129
SM C18:1	-.071	.142	-.019	-.029	-.137	.039	**-.057**	-.182	-.018	-.088	.128	.094
Val	.084	.020	.188	.014	.160	.019	**-.076**	.095	-.169	.103	.055	-.136

Supplementary Table S3. Selected PCA loading scores for females of age-matched mouse strains. Metabolites with a correlation bigger than 0.9 with principle components 1, 2, or 3 at each individual sampling time point were selected. Loading scores with a correlation bigger than 0.9 are written in bold. Loading scores of metabolites selected for further analyses (see Figure 4B) are written in italics. Metabolites selected for further analysis at all four sampling time points are marked with an asterisk.

	6 weeks			10 weeks			14 weeks			20 weeks		
**Metabolite**	**PC1**	**PC2**	**PC3**	**PC1**	**PC2**	**PC3**	**PC1**	**PC2**	**PC3**	**PC1**	**PC2**	**PC3**
Orn	.060	-.118	.163	.055	.112	.149	.032	.125	-.036	*.021*	*.047*	***.234***
PC aa C32:0	-.113	.021	.058	-.108	.005	.045	**-.124**	.031	.015	-.114	.070	-.001
PC aa C36:4	-.119	.019	.055	-.131	-.075	-.032	**-.124**	.055	-.017	-.119	-.081	.020
PC aa C38:0	***-.128***	*.020*	*.043*	-.133	-.023	.000	-.117	-.073	.050	**-.124**	.021	-.049
PC aa C40:2	-.109	-.093	.082	*-.072*	*.144*	*.053*	***-.123***	*-.034*	*-.056*	**-.123**	.012	.056
PC aa C40:3	-.116	-.069	.072	-.107	.047	.080	-.120	.036	-.042	***-.121***	*-.014*	*.060*
PC aa C40:4	***-.126***	*.003*	*-.025*	-.128	-.053	.064	***-.128***	*.025*	*-.015*	***-.124***	*-.060*	*.044*
PC aa C40:5	***-.121***	*.046*	*-.015*	-.114	-.117	.030	*-.097*	*.135*	*.032*	-.106	-.095	.078
PC aa C42:0	***-.123***	*-.027*	*.071*	-.113	.082	-.034	-.118	-.041	.021	-.120	.026	-.037
PC aa C42:1	***-.125***	*-.019*	*.070*	-.091	.091	.030	-.123	-.018	.027	***-.124***	*.034*	*-.012*
PC aa C42:2	-.102	-.098	.115	*-.054*	*.150*	*.079*	***-.125***	*-.031*	*-.021*	**-.126**	.001	.021
PC aa C42:4	***-.126***	*-.001*	*.067*	-.136	-.002	.010	***-.129***	*-.031*	*.023*	***-.127***	*-.002*	*-.017*
PC aa C42:5	***-.124***	*.033*	*.069*	*-.122*	*-.090*	*.076*	***-.128***	*.020*	*.045*	**-.129**	-.035	.012
PC ae C34:0	-.120	.013	-.011	-.119	.045	-.017	**-.123**	-.046	.022	-.087	.116	-.084
PC ae C36:1	-.093	-.141	-.050	-.053	.161	.082	-.098	-.060	-.140	**-.121**	.039	.000
PC ae C36:4	***-.122***	*.048*	*.017*	*-.131*	*.017*	*-.052*	-.113	-.073	-.014	-.113	.073	-.069
PC ae C38:0	-.111	.028	-.015	-.116	-.104	.068	-.119	.065	.057	**-.124**	-.060	-.026
PC ae C38:3	-.115	-.104	-.023	-.103	.123	.086	-.118	-.007	-.093	***-.125***	*.021*	*.042*
PC ae C38:4*	***-.125***	*.043*	*-.002*	*-.136*	*-.018*	*-.089*	***-.123***	*-.074*	*.033*	***-.121***	*.007*	*-.048*
PC ae C38:5	***-.122***	*.066*	*-.007*	*-.134*	*-.012*	*-.019*	***-.124***	*-.024*	*.005*	**-.127**	-.010	.006
PC ae C38:6	***-.126***	*-.009*	*.032*	-.136	.016	-.078	*-.122*	*-.048*	*.055*	-.117	.071	-.065
PC ae C40:0	***-.122***	*.046*	*.008*	*-.090*	*-.150*	*.055*	-.118	.072	.064	-.115	-.082	.043
PC ae C40:2	-.120	-.091	.022	*-.097*	*.135*	*.042*	***-.127***	*-.012*	*-.025*	***-.122***	*.050*	*.048*
PC ae C40:3	-.117	-.096	.015	*-.096*	*.140*	*.025*	***-.127***	*-.026*	*-.055*	***-.127***	*.042*	*.031*
PC ae C40:4	***-.127***	*-.019*	*.001*	*-.137*	*.019*	*-.079*	-.121	-.084	-.006	***-.127***	*.012*	*-.005*
PC ae C40:5*	***-.129***	*.033*	*.037*	***-.142***	*-.054*	*.017*	***-.127***	*.051*	*.042*	***-.127***	*-.025*	*.017*
PC ae C40:6	***-.127***	*.030*	*.015*	*-.136*	*-.032*	*-.097*	-.121	-.053	.092	-.119	.022	-.077
PC ae C42:0	**-.124**	.004	-.026	-.101	-.128	.054	*-.122*	*.047*	*.029*	-.114	-.097	.036
PC ae C42:2	**-.121**	.032	.056	-.101	-.076	.157	*-.093*	*.137*	*.016*	-.118	-.074	.071
PC ae C44:3	-.108	.020	.125	-.012	-.031	.157	-.079	.104	.007	***-.124***	*-.016*	*-.021*
PC ae C44:4	-.116	.024	.104	-.091	-.035	.135	*-.122*	*.058*	*.038*	***-.125***	*-.028*	*.029*
PC ae C44:6	***-.124***	*-.010*	*.071*	-.131	-.002	-.034	-.122	.007	.008	-.109	.081	.008
SM C16:1	-.117	-.069	-.022	***-.138***	*.031*	*-.085*	-.122	-.068	-.004	-.075	.159	-.077

Supplementary Table S4. Selected PCA loading scores for males of age-matched mouse strains. Metabolites with a correlation bigger than 0.9 with principle components 1, 2, or 3 at each individual sampling time point were selected. Loading scores with a correlation bigger than 0.9 are written in bold. Loading scores of metabolites selected for further analyses (see Figure 4B) are written in italics. Metabolites selected for further analysis at all four sampling time points are marked with an asterisk.

	**6 weeks**			**10 weeks**			**14 weeks**			**20 weeks**		
**Metabolite**	**PC1**	**PC2**	**PC3**	**PC1**	**PC2**	**PC3**	**PC1**	**PC2**	**PC3**	**PC1**	**PC2**	**PC3**
lysoPC a C16:1	*.076*	*.156*	*-.052*	*.059*	*-.135*	*-.151*	*.029*	***-.179***	*-.041*	.027	.124	.143
lysoPC a C17:0	***-.121***	*.051*	*.013*	***-.136***	*-.029*	*-.008*	-.120	.062	-.001	-.107	-.089	-.073
lysoPC a C18:0	-.108	.050	-.010	-.114	-.078	-.069	-.105	-.060	.089	**-.121**	-.009	-.011
lysoPC a C18:1	.064	.148	-.011	*.028*	*-.142*	*-.164*	*-.019*	***-.182***	*-.037*	-.026	.129	.114
lysoPC a C20:3	.090	.097	.014	*.030*	*-.147*	*-.151*	-.023	**-.174**	-.009	-.030	.154	.088
PC aa C24:0	**-.117**	.044	-.006	-.006	-.016	-.119	-.117	.005	.009	*-.085*	*.044*	*.132*
PC aa C32:2	.050	.156	-.111	*.015*	*-.149*	*-.119*	*.005*	***-.173***	*-.104*	.005	.148	.159
PC aa C34:4	*.010*	***.197***	*-.095*	-.037	-.166	-.064	*-.002*	***-.182***	*-.055*	*-.057*	*.171*	*-.007*
PC aa C36:5	.047	.164	-.104	*.001*	*-.163*	*-.113*	*-.015*	***-.173***	*-.107*	-.058	.158	.103
PC aa C38:0	**-.121**	.027	-.042	**-.145**	.022	.010	*-.124*	*.061*	*-.047*	***-.120***	*-.015*	*-.090*
PC aa C38:6	**-.118**	.036	-.044	***-.139***	*-.052*	*.021*	*-.124*	*-.064*	*.032*	-.108	.087	-.082
PC aa C40:2	***-.125***	*.004*	*-.024*	-.124	.074	-.024	*-.126*	*.067*	*-.045*	***-.119***	*-.015*	*.057*
PC aa C40:3	-.116	.047	-.042	*-.132*	*-.018*	*-.047*	***-.131***	*-.023*	*.021*	-.112	.021	.097
PC aa C40:4	-.112	.067	-.023	*-.134*	*-.071*	*.009*	***-.129***	*-.046*	*.032*	-.115	.079	.004
PC aa C40:5	***-.120***	*.038*	*.014*	*-.128*	*-.088*	*.030*	-.119	-.091	.036	-.108	.097	-.043
PC aa C40:6	-.092	-.066	-.036	***-.142***	*-.013*	*.052*	-.115	.001	.108	-.110	.071	-.088
PC aa C42:0	***-.126***	*.009*	*.016*	-.089	.111	-.017	-.124	.041	.002	*-.115*	*-.039*	*-.012*
PC aa C42:1	***-.122***	*.002*	*.022*	-.094	.100	-.040	-.119	.041	-.028	**-.120**	-.031	-.021
PC aa C42:2	**-.124**	-.010	-.021	-.104	.114	-.045	-.121	.067	-.052	***-.117***	*-.022*	*.075*
PC aa C42:4	**-.122**	.031	-.016	***-.142***	*.013*	*.035*	***-.134***	*.024*	*.000*	***-.122***	*.002*	*-.016*
PC aa C42:5	**-.118**	.062	-.003	***-.136***	*-.049*	*.017*	***-.133***	*-.035*	*.027*	***-.117***	*.053*	*.010*
PC aa C42:6	-.110	.044	-.059	***-.141***	*-.022*	*.046*	***-.128***	*.036*	*.032*	***-.118***	*.037*	*-.028*
PC ae C32:1	***-.121***	*-.030*	*.007*	-.085	.139	-.025	-.092	.103	-.049	-.106	-.086	.039
PC ae C34:3	-.070	-.130	-.042	*-.014*	***.180***	*-.027*	*-.042*	*.153*	*-.139*	*-.076*	*-.146*	*.019*
PC ae C36:3	-.078	.120	-.078	-.079	.008	-.110	-.094	-.020	-.187	**-.117**	.001	-.020
PC ae C36:5	***-.121***	*.028*	*.025*	-.104	.095	.034	*-.082*	*.091*	*-.146*	-.106	-.041	-.130
PC ae C38:3	***-.126***	*.024*	*.012*	-.105	.032	-.124	-.126	-.011	-.083	**-.125**	.006	.038
PC ae C38:4*	***-.122***	*.059*	*.019*	***-.136***	*-.046*	*.072*	***-.136***	*-.009*	*-.007*	*-.110*	*.043*	*-.137*
PC ae C38:6*	***-.128***	*.002*	*.003*	*-.133*	*.068*	*.059*	***-.128***	*.051*	*-.058*	***-.117***	*-.034*	*-.100*
PC ae C40:2	***-.124***	*-.010*	*.001*	-.106	.095	-.076	***-.130***	*.036*	*-.044*	***-.122***	*-.034*	*.048*
PC ae C40:3	***-.127***	*.012*	*-.008*	-.089	.092	-.113	**-.133**	.000	.002	***-.121***	*-.020*	*.048*
PC ae C40:4*	***-.121***	*.058*	*.009*	***-.140***	*-.044*	*.053*	***-.134***	*.004*	*.003*	***-.118***	*.046*	*-.085*
PC ae C40:5	***-.124***	*.046*	*.035*	***-.138***	*-.060*	*-.007*	***-.134***	*-.051*	*-.009*	***-.120***	*.037*	*-.074*
PC ae C40:6*	***-.128***	*-.004*	*.009*	***-.140***	*.022*	*.079*	***-.126***	*.062*	*.000*	***-.112***	*-.026*	*-.140*
PC ae C44:3	***-.122***	*.044*	*.016*	***-.099***	*-.034*	*-.062*	***-.126***	*-.023*	*-.011*	***-.120***	*.015*	*.011*
PC ae C44:4	***-.117***	*.063*	*-.032*	***-.112***	*-.067*	*-.060*	***-.119***	*-.052*	*-.064*	***-.113***	*.059*	*.047*
PC ae C44:6	***-.126***	*.019*	*.003*	***-.131***	*.044*	*-.006*	***-.122***	*.023*	*-.059*	***-.115***	*-.053*	*-.017*
SM (OH) C22:2	***-.119***	*-.042*	*.070*	***-.138***	*.037*	*.019*	***-.123***	*.053*	*.011*	***-.078***	*-.128*	*-.088*
SM C16:1	***-.116***	*-.075*	*.054*	***-.137***	*.051*	*.040*	***-.124***	*.057*	*.037*	***-.117***	*-.058*	*-.029*

Supplementary Table S5. Fold changes of selected metabolites showing overlap in age regulation among female mice of different strains. Listed are fold changes in levels of plasma metabolites that were found to be age dependently regulated in three of the four analyzed mouse strains. Fold changes were calculated for the time periods 6 to 10, 10 to 14, and 14 to 20 weeks of age. Changes were tested for significance using Wilcoxon rank sum tests. P-values were corrected for multiple testing using the Benjamini & Hochberg procedure and are given in brackets. * p ≤ 0.05, ** p ≤ 0.01, *** p ≤ 0.001, n.s. not significant.

	**Age [weeks]**	**lysoPC a C17:0**	**lysoPC a C18:0**	**PC aa C42:2**	**PC aa C42:5**	**PC ae C40:2**	**PC ae C40:5**	**PC ae C42:3**	**PC ae C44:3**
**129S1**	6 vs 10	1.10 (n.s.)	1.02 (n.s.)	1.01 (n.s.)	0.96 (n.s.)	1.02 (n.s.)	1.08 (n.s.)	0.87 (n.s.)	0.97 (n.s.)
	10 vs 14	1.31 (*)	1.12 (n.s.)	1.20 (**)	1.25 (**)	1.10 (n.s.)	1.11 (*)	1.40 (**)	1.25 (*)
	14 vs 20	0.91 (n.s.)	0.89 (n.s.)	0.93 (n.s.)	0.89 (n.s.)	0.91 (n.s.)	1.05 (n.s.)	1.06 (n.s.)	1.52 (*)
**B6J**	6 vs 10	0.97 (n.s.)	0.85 (n.s.)	1.09 (n.s.)	0.95 (n.s.)	1.07 (n.s.)	1.04 (n.s.)	0.85 (n.s.)	1.28 (*)
	10 vs 14	1.46 (***)	1.24 (*)	1.21 (n.s.)	1.08 (n.s.)	0.99 (n.s.)	0.96 (n.s.)	1.07 (n.s.)	0.91 (n.s.)
	14 vs 20	0.60 (***)	0.68 (**)	0.60 (*)	0.75 (*)	0.74 (**)	0.85 (**)	0.89 (n.s.)	0.79 (*)
**B6NTa**	6 vs 10	1.27 (*)	1.14 (n.s.)	1.05 (n.s.)	1.22 (*)	1.02 (n.s.)	1.21 (*)	1.32 (**)	1.18 (n.s.)
	10 vs 14	0.85 (n.s.)	0.83 (n.s.)	0.95 (n.s.)	0.75 (*)	1.00 (n.s.)	0.93 (n.s.)	0.92 (n.s.)	1.15 (n.s.)
	14 vs 20	1.44 (*)	1.32 (*)	0.56 (***)	0.71 (n.s.)	0.79 (*)	0.72 (*)	0.68 (*)	0.37 (***)
**C3Fe**	6 vs 10	0.84 (**)	0.94 (n.s.)	0.59 (**)	0.88 (n.s.)	0.74 (**)	0.96 (n.s.)	0.97 (n.s.)	0.62 (**)
	10 vs 14	1.16 (*)	1.19 (*)	1.58 (***)	0.97 (n.s.)	1.57 (**)	1.18 (*)	1.06 (n.s.)	1.64 (**)
	14 vs 20	0.63 (***)	0.66 (***)	0.96 (n.s.)	1.12 (n.s.)	0.81 (n.s.)	1.00 (n.s.)	1.20 (n.s.)	0.93 (n.s.)

Supplementary Table S6. Fold changes of selected metabolites showing overlap in age regulation among male mice of different strains. Listed are fold changes in levels of plasma metabolites that were found to be age dependently regulated in three of the four analyzed mouse strains. Fold changes were calculated for the time periods 6 to 10, 10 to 14, and 14 to 20 weeks of age. Changes were tested for significance using Wilcoxon rank sum tests. P-values were corrected for multiple testing using the Benjamini & Hochberg procedure and are given in brackets. * p ≤ 0.05, ** p ≤ 0.01, *** p ≤ 0.001, n.s. not significant.

	**Age [weeks]**	**lysoPC a C20:4**	**PC aa C34:2**	**PC aa C36:2**	**PC aa C36:4**	**PC ae C38:1**	**PC ae C38:2**	**SM C18:1**	**SM C24:1**
**129S1**	6 vs 10	0.65 (**)	1.01 (n.s.)	1.04 (n.s.)	1.01 (n.s.)	1.04 (n.s.)	1.49 (**)	0.97 (n.s.)	0.83 (*)
	10 vs 14	1.07 (n.s.)	0.95 (n.s.)	0.92 (*)	0.84 (**)	1.36 (*)	1.17 (n.s.)	0.76 (**)	0.86 (n.s.)
	14 vs 20	0.87 (n.s.)	0.94 (n.s.)	1.00 (n.s.)	1.03 (n.s.)	0.99 (n.s.)	1.13 (n.s.)	1.19 (*)	1.09 (n.s.)
**B6J**	6 vs 10	0.68 (**)	1.18 (**)	1.25 (**)	1.16 (**)	1.61 (**)	1.50 (**)	0.87 (**)	0.76 (**)
	10 vs 14	0.75 (n.s.)	0.87 (*)	0.90 (*)	0.81 (**)	1.31 (**)	1.27 (**)	0.80 (**)	1.00 (n.s.)
	14 vs 20	1.25 (n.s.)	0.91 (**)	0.81 (**)	0.99 (n.s.)	0.51 (**)	0.65 (**)	1.11 (n.s.)	1.10 (n.s.)
**B6NTa**	6 vs 10	0.83 (n.s.)	0.93 (n.s.)	0.96 (n.s.)	0.93 (n.s.)	1.88 (**)	1.59 (**)	0.67 (*)	0.74 (*)
	10 vs 14	1.10 (n.s.)	1.18 (*)	1.12 (*)	1.07 (n.s.)	0.98 (n.s.)	1.18 (n.s.)	0.90 (n.s.)	1.03 (n.s.)
	14 vs 20	0.68 (**)	1.11 (n.s.)	1.09 (n.s.)	0.98 (n.s.)	1.07 (n.s.)	1.04 (n.s.)	0.89 (n.s.)	0.75 (**)
**C3Fe**	6 vs 10	1.18 (**)	1.06 (n.s.)	1.12 (n.s.)	1.09 (n.s.)	0.70 (**)	1.06 (n.s.)	0.82 (**)	0.80 (**)
	10 vs 14	0.87 (*)	1.02 (n.s.)	1.04 (n.s.)	0.99 (n.s.)	1.72 (**)	1.26 (**)	0.96 (n.s.)	0.91 (n.s.)
	14 vs 20	0.91 (n.s.)	1.22 (**)	1.18 (**)	1.16 (**)	1.29 (*)	1.46 (**)	0.87 (*)	0.81 (**)
